# Ancestral and novel roles of Pax family genes in mollusks

**DOI:** 10.1186/s12862-017-0919-x

**Published:** 2017-03-16

**Authors:** Maik Scherholz, Emanuel Redl, Tim Wollesen, André Luiz de Oliveira, Christiane Todt, Andreas Wanninger

**Affiliations:** 1 Department of Integrative Zoology, Faculty of Life Sciences, University of Vienna, Althanstraße 14, 1090 Vienna, Austria; 2 University Museum of Bergen, University of Bergen, Allégaten 41, 5007 Bergen, Norway

**Keywords:** Mollusca, Aculifera, Lophotrochozoa, Tetraneural nervous system, Pax genes, Gene expression, Neurogenesis, Development, Evolution, EvoDevo

## Abstract

**Background:**

Pax genes are transcription factors with significant roles in cell fate specification and tissue differentiation during animal ontogeny. Most information on their tempo-spatial mode of expression is available from well-studied model organisms where the Pax-subfamilies *Pax2/5/8, Pax6*, and *Paxα/β* are mainly involved in the development of the central nervous system (CNS), the eyes, and other sensory organs. In certain taxa, *Pax2/5/8* seems to be additionally involved in the development of excretion organs. Data on expression patterns in lophotrochozoans, and in particular in mollusks, are very scarce for all the above-mentioned Pax-subfamilies, which hampers reconstruction of their putative ancestral roles in bilaterian animals. Thus, we studied the developmental expression of *Pax2/5/8, Pax6*, and the lophotrochozoan-specific *Paxβ* in the worm-shaped mollusk *Wirenia argentea*, a member of Aplacophora that together with Polyplacophora forms the Aculifera, the proposed sister taxon to all primarily single-shelled mollusks (Conchifera).

**Results:**

All investigated Pax genes are expressed in the developing cerebral ganglia and in the ventral nerve cords, but not in the lateral nerve cords of the tetraneural nervous system. Additionally, *Pax2/5/8* is expressed in epidermal spicule-secreting or associated cells of the larval trunk and in the region of the developing protonephridia. We found no indication for an involvement of the investigated Pax genes in the development of larval or adult sensory organs of *Wirenia argentea*.

**Conclusions:**

*Pax2/5/8* seems to have a conserved role in the development of the CNS, whereas expression in the spicule-secreting tissues of aplacophorans and polyplacophorans suggests co-option in aculiferan skeletogenesis. The *Pax6* expression pattern in Aculifera largely resembles the common bilaterian expression during CNS development. All data available on *Paxβ* expression argue for a common role in lophotrochozoan neurogenesis.

**Electronic supplementary material:**

The online version of this article (doi:10.1186/s12862-017-0919-x) contains supplementary material, which is available to authorized users.

## Background

The morphological diversity of the phylum Mollusca is represented by eight recent clades, including the well-known gastropods, bivalves, and cephalopods. This bodyplan plasticity renders mollusks an ideal target for evolutionary and developmental studies. Although numerous aspects of intra-molluscan relationships are still controversially discussed, recent phylogenomic analyses show a basal dichotomy comprising the monophyletic Aculifera (including the eight-shelled Polyplacophora and the shell-less, spicule-bearing aplacophoran clades Neomeniomorpha or Solenogastres and Chaetodermomorpha or Caudofoveata) and the primarily single-shelled Conchifera (Monoplacophora, Gastropoda, Cephalopoda, Scaphopoda, Bivalvia) [1–3]. The vermiform Neomeniomorpha represents one of the least investigated molluscan taxa, although recent comparative studies have demonstrated its importance to reconstruct ancestral aculiferan traits [4, 5].

**Fig. 1:**
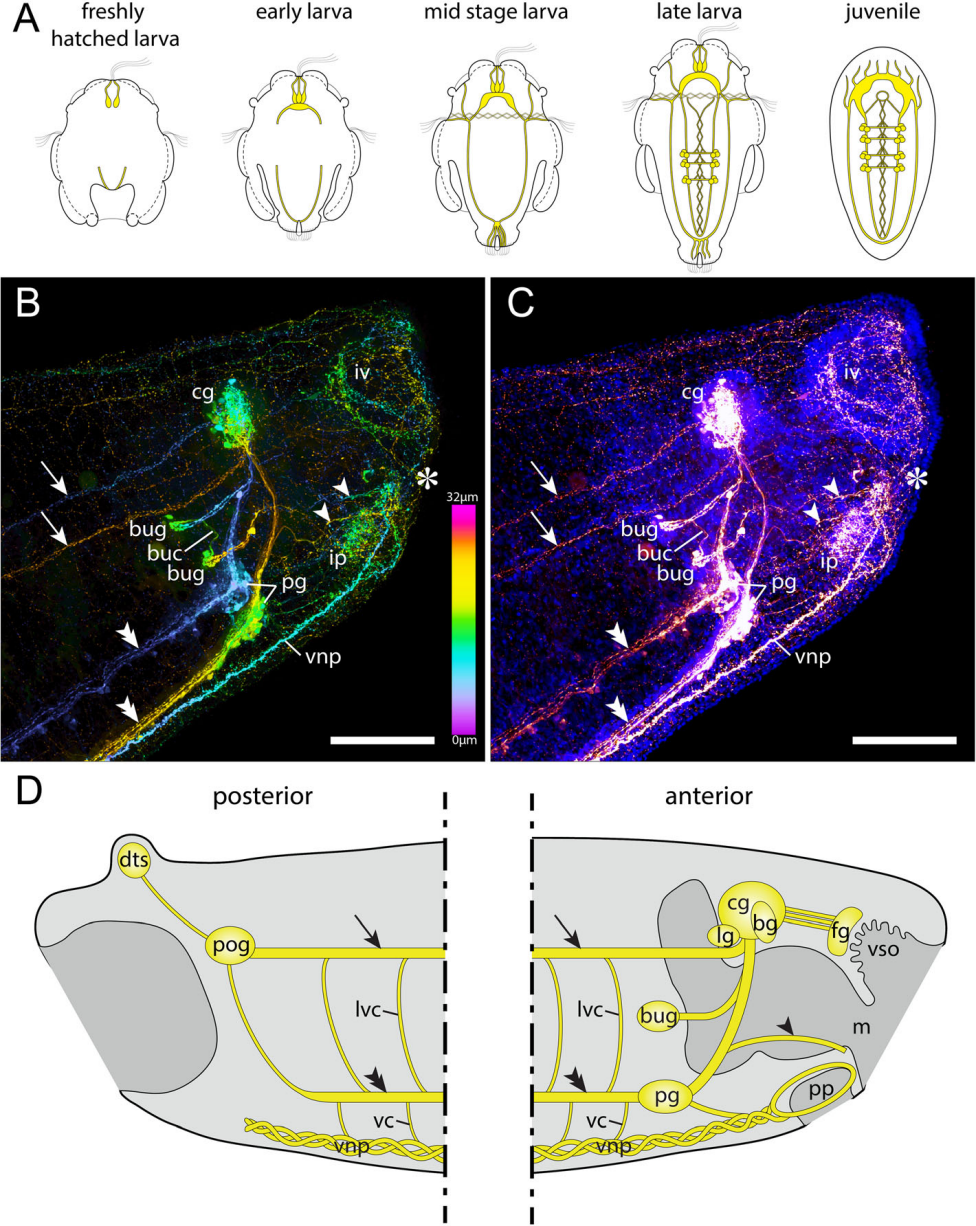
Summary of neomeniomorph neurogenesis and adult neuroanatomy. **a** Schematic representation of neomeniomorph neurogenesis from the freshly hatched test cell larva until the juvenile stage. Anterior faces up. Neural structures are drawn in yellow. Reconstruction based on Redl et al. (2014). **b** Confocal scan (maximum intensity projection, color depth coding) of the adult CNS, anterior region. Anterior to the right. Anti-serotonin (5-HT) staining. **c** Same confocal scan (maximum intensity projection) as in (**b**), but with additional nucleic acid staining (DAPI) to reveal nuclei of the cerebral ganglia. **d** Schematic representation of the posterior and anterior major elements of the adult nervous system of *Wirenia argentea* based on (**b**) and (**c**) as well as Todt et al. (2008). Abbreviations: vestibular (atrial) sense organ (vso); basal ganglion (bg); buccal commissure (buc); buccal ganglion (bug); cerebral ganglion (cg); dorsoterminal sense organ (dts); frontal ganglia (fg); innervation of vestibular (atrial) sense organ (iv); innervation of pedal pit (ip); lateral ganglion (lg); lateroventral commissure (lvc); mouth (m); pedal ganglion (pg); pedal pit (pp); posterior ganglion (pog); ventral commissure (vc); ventral neural plexus (vnp); nerves of pedal pit (arrowhead); lateral (visceral) nerve cord (arrow); ventral (pedal) nerve cord (double arrowhead); mouth opening (asterisk). Scale bars: 100 μm

The adult neomeniomorph nervous system reflects the specific molluscan condition of an esophageal nerve ring, which includes a cerebral ganglion as well as a paired pedal ganglion. Two lateral (visceral) nerve cords emanate from the cerebral ganglion, while the pedal ganglia give rise to a pair of ventral (pedal) nerve cords. Together, these four longitudinal nerve cords form the molluscan tetraneural nervous system [6–10] (see also Fig. 1). During early stages of neomeniomorph neurogenesis the subsidence of epidermal cells results in two lateral depressions of the anterior larval episphere (often termed “ectodermal cerebral depressions”), which give rise to the anlagen of the cerebral ganglion [11, 12]. Before the tetraneural condition is established, two longitudinal neurite bundles emerge simultaneously from both the anterior and the posterior pole and subsequently fuse in the region of the prototroch, the locomotive ciliary band of the free-swimming larva [13] (Fig. 1a). This formation of an intermediate stage with a single pair of nerve cords has recently been interpreted as a putative ancestral feature of spiralian neurogenesis [13].

Adult neomeniomorphs lack eyes but do exhibit several sensory organs, including a vestibular (atrial) sense organ and a dorsoterminal sense organ that is often considered a homolog of the osphradium of other molluscan clades [6, 14–16] (Fig. 1b, c, d). Additionally, some neomeniomorphs exhibit a pedal commissural sac that was suggested to serve as a geosensory organ [17]. Similar to many other spiralians, neomeniomorph larvae exhibit an apical organ with a ciliary tuft [13, 18]. Interestingly, and in contrast to the aplacophoran clades, representatives of the Polyplacophora exhibit ventrally positioned posttrochal larval eyes, which are lost some time after metamorphosis.

The highly conserved paired box (Pax) genes encode for a family of metazoan transcription factors, which are crucial for cell fate specification and tissue differentiation and are known to be involved in the development of excretory organs, myogenesis, neurogenesis, biomineralisation processes (skeletogenesis), and the development of visual and geosensory systems in numerous bilaterians (e.g., [19–21]). Although neomeniomorph larvae lack a number of sensory organs such as eyes and statocysts, they possess Pax genes whose orthologs have a conserved function in neurogenesis and sensory organ development. This poses the question as to where these genes are expressed during the ontogeny of neomeniomorphs.

*Pax2/5/8* is known to be involved in the development of excretory systems of certain annelids, onychophorans, vertebrates, as well as in the formation of auditory/geosensory systems and in establishing the midbrain/hindbrain boundary of vertebrates as well as the deutocerebrum/tritocerebrum boundary of ecdysozoans [22–24]. The developmental expression and putative function of *Pax2/5/8* has also been studied in a few lophotrochozoan taxa [25–28]. In mollusks, *Pax2/5/8* is expressed during development of multimodal sensory systems of gastropods [25], polyplacophorans, and cephalopods [27]. Furthermore, *Pax2/5/8* is expressed in the mantle of gastropods, polyplacophorans, bivalves, and cephalopods, which is known to be rich in sensory structures [25, 27]. In the polychaete annelid *Platynereis dumerilii Pax2/5/8* expression was found during development of photoreceptor cells of regenerating segments [29], while in the leech *Helobdella austinensis Pax2/5/8* expression is confined to the neuroectoderm of the developing ventral nerve cords and to the developing nephridia [26]. A recent study of *Pax6* and *Pax2/5/8* in two brachiopods suggested that these genes have a putative role as regulators of the segment polarity gene *engrailed*, although further details remain vague [28]. *Pax6* is generally involved in the development of the bilaterian central nervous system (CNS) and is a key player in eye gene regulatory networks of most bilaterians, including cephalopod and polyplacophoran mollusks [30–32]. *Paxβ* represents the lophotrochozoan-specific ortholog of the recently discovered Paxα/β subfamily and has hitherto been exclusively studied in the leech *Helobdella austinensis* [33–35]. Late embryos of *H. austinensis* show broad, segmentally restricted mesodermal expression as well as expression in the CNS and eyes during organogenesis [33]. So far, the expression pattern of *Paxα* (the deuterostome and ecdysozoan *Paxβ* ortholog) is exclusively known from the onychophoran *Euperipatoides rowelli* [35].

In order to further assess putative ancestral versus novel roles of these important developmental regulators, we here provide the first detailed study of selected Pax family genes in a hitherto largely neglected but evolutionarily highly important molluscan clade, the Neomeniomorpha.

## Methods

### Animal cultures

Adult and developmental stages of the neomeniomorph *Wirenia argentea* Odhner, 1921 were collected, maintained, and reared from January to May 2012, November 2012 to February 2013, and from November to December 2013, respectively, following Redl et al. [13] with the following modifications during the last season: The sediment sample was kept in 20μm-filtered and UV-sterilized sea water with a salinity of 35‰ (FSSW) precooled to 4°C. Every four days the adult specimens were transferred to clean plastic jars with fresh FSSW, which increased egg laying productivity. The freshly laid eggs were transferred into clean plastic jars with FSSW and kept under the same conditions as the adults. After hatching *Wirenia* develops via the so-called pericalymma or test cell larva. Herein, age of the larvae is given in days post hatching (dph). We fixed four morphologically distinguishable stages: freshly hatched test cell larva (0–1 dph), early test cell larva (6–7 dph), mid-stage test cell larva (10–11 dph), and late test cell larva (14–15 dph), whereby each stage encompasses a developmental time range of approximately 24 h.

### Immunochemistry

Relaxation, fixation, storage as well as all immunocytochemical procedures followed standard protocols as described in detail in Redl et al. [13] and Scherholz et al. [5], respectively.

### RNA extraction and fixation of animals

All molecular biological procedures, ranging from RNA extraction until the end of the *in situ* hybridization protocol, were conducted using RNase-free (or diethylpyrocarbonate (DEPC)-treated) water. Total RNA was extracted from larvae using the Qiagen RNeasy Mini Kit (Qiagen, Venlo, The Netherlands) with the QIAshredder homogenizer (Qiagen). Prior to RNA extraction the larval material was either shock frozen on dry ice or conserved in RNAlater. Additional RNA was extracted from adult specimens. In order to separate animal tissue from the gut content and to avoid contamination, all adult specimens were starved for at least two weeks prior to RNA extraction. Adult RNA was extracted using TRI reagent (Sigma-Aldrich) according to the manufacturer’s instructions with the optional centrifugation step after homogenization and the following modifications: The animals were shock frozen with dry ice immediately before homogenization and RNA precipitation was performed with a 1:1 mixture of isopropanol and a high salt precipitation solution containing 0.8mol/l trisodium citrate dihydrate and 1.2mol/l sodium chloride. RNA pellets were dissolved in water and stored at −80°C.

Advanced larval stages were relaxed prior to fixation for 20 to 30 min at 4°C by adding a 3.2% magnesium chloride solution. Developmental stages were fixed for in situ hybridization with 4% paraformaldehyde (PFA) in 0.1M MOPS buffer (with 0.5M/l NaCl, 2mM/l MgSO_4_, and 1mM/l EGTA added) for 45 min at room temperature (RT). Fixed larvae were stepped into precooled (4°C) or prechilled (−20°C) 75% EtOH, washed three times for a total period of 15–30 min in precooled (4°C) or for 45 min to 1.5 h in prechilled (−20°C) 75% EtOH, and stored in fresh 75% EtOH at −20 °C.

### RNAseq and transcriptome assembly

Larval and adult RNA samples were pooled and sequenced by Illumina technology (Eurofins, Ebersberg, Germany). Sequencing and bioinformatic processing of the resulting paired-end libraries as well as the transcriptome assembly were performed as described in Redl et al. [36].

### Gene identification and orthology assignment

We identified candidate genes in our transcriptome database by reciprocal BLAST [37] searches using bilaterian Pax gene sequences from NCBI GenBank as queries. Nucleotide sequences of the best-fitting contigs were translated into amino acid sequences using Geneious, Version 6.1.6 (Biomatters, Auckland, New Zealand).

Gene orthology was determined by phylogenetic reconstruction. FASTA-formatted files were generated with the inferred amino acid sequences for cloned genes and representative homologs from other bilaterian taxa (see Table 1 for accession numbers). Sequence alignment was performed with the online version of MAFFT (http://www.ebi.ac.uk/Tools/msa/mafft/; [38]) with the following modifications to the standard setting: MAXITERATE → 100 (long run); PERFORM FFTS → localpair. The resulting alignment was checked and manually edited using BioEdit [39] to remove non-conserved regions. The phylogenetic analysis was carried out with the Bayesian phylogenetic program MrBayes v.3.2.6 [40]. A specified evolutionary model was determined using Akaike Information Criterion (AIC) as implemented in ProtTest 3 [41]. The following parameters were employed in MrBayes: Jones-Taylor-Thornton model of amino-acid substitution [42]; six rates categories for the gamma distribution; 30,000,000 generations; sample frequency 1,000. After the removal of 25% of the sampled trees as burn-in, the final phylogenetic tree was created and subsequently edited with FigTree v1.4.2 (http://tree.bio.ed.ac.uk/software/figtree/; [43]). The resulting illustration was modified with Adobe Illustrator CC 2015 (Adobe Systems, San José, California, USA).

**Table 1:** GenBank accession numbers of genes used in the phylogenetic analysis

	Species	Phylum	Gene	GenBank accession number
*	*Wirenia argentea*	Mollusca	*Paxβ*	[KY488206]
*	*Acanthochitona crinita*	Mollusca	*Paxβ*	[KY488203]
	*Aplysia californica*	Mollusca	*Paxβ*	[DAA12510]
	*Lottia gigantea*	Mollusca	*Paxβ*	[DAA12512]
	*Capitella teleta*	Annelida	*Paxβ*	[DAA12511]
	*Helobdella austinensis*	Annelida	*Paxβ2*	[ABQ45871]
	*Schmidtea mediterranea*	Platyhelminthes	*Paxβ2*	[DAA12514]
	*Euperipatoides rowelli*	Onychophora	*Paxα*	[AJG44471]
	*Tribolium castaneum*	Arthropoda	*Pox-neuro*	[EFA07416]
	*Drosophila melanogaster*	Arthropoda	*Pox-neuro*	[AAA28832]
	*Saccoglossus kowalevskii*	Hemichordata	*Pox-neuro*	[NP_001158393]
*	*Wirenia argentea*	Mollusca	*Pax2/5/8*	[KY488205]
	*Acanthochitona crinita*	Mollusca	*Pax2/5/8*	[ALM30867]
	*Platynereis dumerilii*	Annelida	*Pax2/5/8*	[AGC12568]
	*Crassostrea gigas*	Mollusca	*Pax2A*	[EKC36239]
	*Saccoglossus kowalevskii*	Hemichordata	*Pax2/5/8*	[ADB22664]
	*Euperipatoides rowelli*	Onychophora	*Pax2/5/8*	[AJG44467]
	*Gallus gallus*	Chordata	*Pax2*	[NP_990124]
	*Branchiostoma belcheri*	Chordata	*Pax2*	[ABK54277]
	*Tribolium castaneum*	Arthropoda	*Pax2/5/8*	[EFA01334]
	*Ciona intestinalis*	Chordata	*Pax2/5/8*	[NP_001027652]
	*Capitella teleta*	Annelida	*Pax3/7*	[ABC68267]
	*Branchiostoma belcheri*	Chordata	*Pax3/7*	[ABK54280]
	*Octopus bimaculoides*	Mollusca	*Pax3/7*	[ACR19857]
	*Sepia officinalis*	Mollusca	*Pax3/7*	[AHG12548]
	*Crassostrea gigas*	Mollusca	*Pax7*	[EKC41820]
	*Ciona intestinalis*	Chordata	*Pax1/9*	[BAA74829]
	*Terebratalia transversa*	Brachiopoda	*Pax1/9*	[AJV21320]
	*Ptychodera flava*	Hemichordata	*Pax1/9*	[BAA78380]
	*Branchiostoma belcheri*	Chordata	*Pax1/9*	[ABK54274]
*	*Wirenia argentea*	Mollusca	*Pax6*	[KY488204]
*	*Acanthochitona crinita*	Mollusca	*Pax6*	[KY488202]
	*Doryteuthis opalescens*	Mollusca	*Pax6*	[AAB40616]
	*Euprymna scolopes*	Mollusca	*Pax6*	[AF513712]
	*Idiosepius paradoxus*	Mollusca	*Pax6*	[BAM74253]
	*Terebratalia transversa*	Brachiopoda	*Pax6*	[ADZ24784]
	*Lineus sanguineus*	Nemertea	*Pax6*	[CAA64847]
	*Platynereis dumerilii*	Annelida	*Pax6*	[CAJ40659]
	*Saccoglossus kowalevskii*	Hemichordata	*Pax6*	[NP_001158383]

Pax sequences of Neomeniomorpha and Polyplacophora retrieved from our transcriptomic data are labeled by asterisk

### Gene cloning and probe synthesis

Oligonucleotide primers were designed from contigs using Geneious, Version 6.1.6 (Biomatters, Auckland, New Zealand). Thus, the defined gene fragments include the entire sequences of the conserved Paired domain and its flanking 5′ and 3′ ends in case of *War-Pax2/5/8* and *War-Paxβ*, whereas the gene fragment of *War-Pax6* comprises a partial sequence of the conserved prd-class homeodomain (and the flanking 3′ end of this conserved region), which is entirely lacking in the *Paxβ* paralog and partially lacking in the *Pax2/5/8* paralog. Specific primers and fragment lengths of probes are available in Table 2. Primers were synthesized by Invitrogen, Life Technologies. The nucleotide sequences as well as the amino acid sequences of the amplified fragments have been deposited at NCBI GenBank (see Table 1 for accession numbers). PCR amplification was performed on a cDNA library synthesized from combined mixed-stage embryonic and adult total RNA. cDNA was synthesized using a 1^st^ Strand cDNA Synthesis Kit for RT-PCR (AMV) (Roche, Basel, Switzerland). Amplified fragments were cloned into pGEM-T Easy Vector System I (Promega, Madison, WI, USA) and the plasmids were used to transform *E. coli* JM109 Competent Cells (Promega). Plasmids were extracted from minipreps using the QIAprep Spin Miniprep Kit (Qiagen) and sequenced by Microsynth (Vienna, Austria). Obtained sequences were compared to known Pax sequences from NCBI GenBank and to the original contigs of the *Wirenia argentea* transcriptome using the BLAST program and Geneious, Version 6.1.6. The linear template for probe synthesis was generated via standard PCR using GoTaq Flexi DNA Polymerase reagents and M13 forward and reverse primers (FFW 5′-GTTTTCCCAGTCACGACGTT-3′, annealing temperature: 60°C; REV 5′-GACCATGATTACGCCAAGCTA-3′, annealing temperature: 60°C). Amplified products were purified using the GeneJET PCR Purification Kit (Thermo Fisher Scientific, Waltham, MA, USA) and subsequently used as templates for anti-sense RNA probe syntheses. Synthesis reaction was performed using a DIG RNA Labeling Kit (SP6/T7) (Roche Life Science). 2μl of 100mM dithiotreitol (DTT) were added to the transcription reaction and, after the reaction, template DNA was removed by incubation with DNase I, RNase free (Roche Life Science). Precipitation of RNA was done at −80°C. Protector RNase Inhibitor (Roche Life Science) was added after dissolving the RNA pellet in water. RNA probes for in situ hybridization were stored at −80°C.

**Table 2:** Primers used for PCR

Gene	Fragment length (in bases)	Direction	Primer sequence
*War-Pax2/5/8*	426	Forward	CAGATTTTCTGTCGGACTTCCTCTGATGTC
		Reverse	GACCCTAACGGTCACGGAGGAGTAAAC
*War-Pax6*	709	Forward	GAATTTGAGAGGACACATTATCCAGAC
		Reverse	GACATAATATATCCGGATCTCCATATTCG
*War-Paxβ*	667	Forward	GCATGGAAAGGTGAGAGTAGATGATTC
		Reverse	GTAACAGATTCTAACGTGATCAACCAC

### Whole-mount in situ hybridization

Samples stored in 75% EtOH were stepped into 4% PFA in 1x Roti-Stock phosphate buffered saline (pH = 7.4; PBS; Carl Roth) with 0.05M/l EGTA (PPE) and decalcified in PPE for 1h at RT. Subsequently, samples were washed six times for 5 min each in PBS with 0.1% Tween 20 (PBT; Carl Roth) at RT and then heated to 37°C in a water bath during the last washing step. Proteinase K treatment was done with a solution of 10μg/ml Proteinase K (Roche Life Science) in PBT for 10 min at 37°C without agitation. The specimens were then washed twice for 5 min each at RT in PBT, twice for 5 min each in 1% triethanolamine (TEA) in PBT, four times for 5 min each in an instantly made mixture of 0.3% acetic anhydride and 1% TEA in PBT, and again twice for 5 min each in PBT. Afterwards, the samples were postfixed in 4% PFA in PBS for 45 min at RT and washed five times for 5 min each at RT in PBT. Samples were subsequently stepped into the hybridization buffer (HB) consisting of 50% formamide with 0.075M/l trisodium citrate, 0.75M/l sodium chloride, 5mM/l EDTA, 50μg/ml heparin sodium salt (Sigma-Aldrich), 1x Denhardt’s Solution (Carl Roth), 100μg/ml RNA from torula yeast, Type VI (Sigma-Aldrich), and 5% dextran sulfate sodium salt from *Leuconostoc* spp. (Sigma-Aldrich). The samples were then transferred into fresh HB, heated in a water bath to 56°C (in case of the *Pax6* probe) or 60°C (in case of *Pax2/5/8* and *Paxβ*), respectively, and prehybridized at these specific temperatures for 15–20 h. All RNA probes were diluted in HB to a final concentration of 1–2μg/ml, denatured for 10 min at 85°C, and applied to the samples. Hybridization was conducted for 24–26 h at the specific temperatures of 56°C and 60°C, respectively. Then, the samples were kept at hybridization temperature and washed three times for 20 min each in pre-warmed 50% formamide with 0.06M/l trisodium citrate, 0.6M/l sodium chloride, and 0.1% Tween 20, twice for 20 min each in 50% formamide with 0.03M/l trisodium citrate, 0.3M/l sodium chloride, and 0.1% Tween 20, and three times for 15 min each in 50% formamide with 0.015M/l trisodium citrate, 0.15M/l sodium chloride, and 0.1% Tween 20. The samples were then placed at RT to cool down. Afterwards, they were washed thrice for 20 min each at RT in 0.015M trisodium citrate solution with 0.15M/l sodium chloride and 0.1% Tween 20. Subsequently, the samples were washed thrice for 5 min each at RT in 0.1M maleic acid buffer (MAB) with 0.15M/l sodium chloride and 0.1% Tween 20 (pH = 7.5). Blocking of unspecific binding sites was done for 3 h at RT with a 2% solution of Blocking Reagent (Roche Life Science) in MAB. Anti-Digoxigenin (DIG)-AP, Fab fragments (Roche Life Science, Ref. 11093274910) were applied in a 1:2,500 or 1:5,000 dilution in 2% block solution for 13–16 h at 4°C. After incubation, the samples were rinsed eight times for 20 min each at RT in PBT, twice for 5 min each without agitation in 0.1M Tris buffer with 0.1M/l sodium chloride (pH = 9.5; AP buffer) and 0.1% Tween 20, and twice for 10 min each without agitation in AP buffer with 50mM/l magnesium chloride and 0.1% Tween 20. Finally, all samples were transferred into staining buffer (AP buffer with 50mM/l magnesium chloride, 7.5% polyvinyl alcohol, and 20μl/ml NBT/BCIP stock solution (Roche Life Science)). Color reaction took place at 4°C for 3–8 h (depending on probe, probe concentration, and DIG concentration) and was stopped by washing twice in 0.1M glycine in PBT (pH = 2.2) followed by additional two washes in PBT, 5 min each. Stained specimens were then fixed for 12–24 h at 4°C in 4% PFA in PBS, subsequently rinsed four times for at least 5 min each in PBT, and finally stored in PBT at 4°C.

### Mounting and clearing

Larval stages were stepped into deionized water and washed four times for 5 min each in deionized water. Larvae were then stepped into EtOH and washed thrice for 5 min each in 100% EtOH. Next, the larvae were transferred into a 1:1 mixture of benzyl benzoate and benzyl alcohol, and subsequently mounted in this medium on microscope slides. Approximately 250 specimens were processed and investigated in total and 81 (25 with *Pax2/5/8* expression, 31 with *Pax6* expression, and 25 with *Paxβ* expression) were scanned with a confocal microscope.

### Microscopy, 3D rendering, and image processing

Specimens were analyzed and light micrographs were taken on an Olympus BX53 microscope equipped with an Olympus DP73 camera and the software cellSens Standard, Version 1.11 (Olympus Corporation, Shinjuku, Tokyo, Japan). Confocal laser scanning microscopy was conducted using a Leica DMI6000 CFS microscope equipped with a Leica TCS SP5 II scanning system (Leica Microsystems, Wetzlar, Germany) and software LAS AF, Version 2.6.0 or 2.6.3. The autofluorescent signal was scanned in fluorescence mode using a 405 nm laser and gene expression signal was scanned in reflection mode using a 633nm laser (see [44–47]). The obtained confocal image stacks were processed and used to prepare 3D reconstructions of the gene expression signal as well as 3D renderings of larval tissue with Imaris x64, Version 7.3.1 (Bitplane AG, Zurich, Switzerland). The same software was used to create video files of larval stages including gene expression patterns. Generated images were finally processed with Adobe Photoshop CS6 Extended, Version 13.0.1 x64, and Adobe Photoshop CC 2015 (Adobe Systems). Figures and schematic drawings were generated with Adobe Illustrator CS5, Version 15.0.0 and Adobe Illustrator CC 2015, Version 1.0 (Adobe Systems).

## Results

### Larval morphology of the neomeniomorph *Wirenia argentea*

**Fig. 2:**
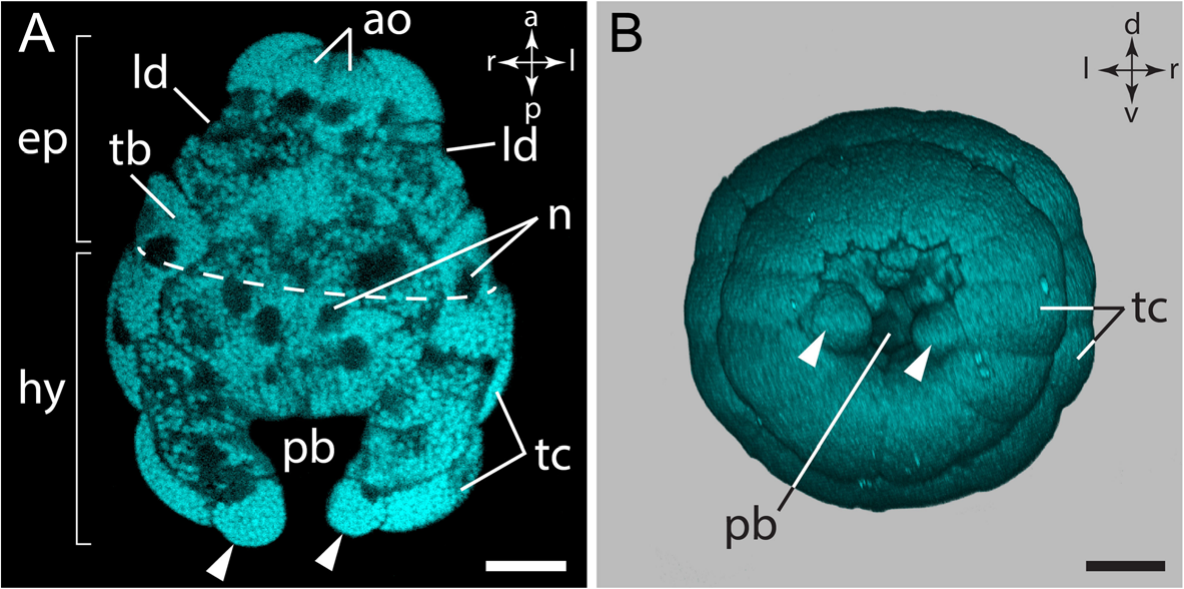
Morphology of a freshly hatched *W. argentea* larva. Dorsal (d)–ventral (v), left (l)–right (r), and anterior (a)–posterior (p) axes indicate the orientation. **a** Optical section of an autofluorescence scan of a freshly hatched test cell larva with anterior facing up. Dashed line indicates the prototroch, which separates the episphere (ep) from the hyposphere (hy). **b** 3D volume rendering based on an autofluorescence scan. Posterior view on the larval hyposphere and the invagination of the pseudo-blastopore of the same specimen as in (**a**). Abbreviations: cells of the apical organ (ao); episphere (ep); hyposphere (hy); lateral depression (ld); nucleus (n); pseudo-blastopore (pb); trochoblast (tb); test cell (tc); the two most posteriorly and laterally positioned test cells (arrowhead). Scale bars: 50 μm

**Fig. 3:**
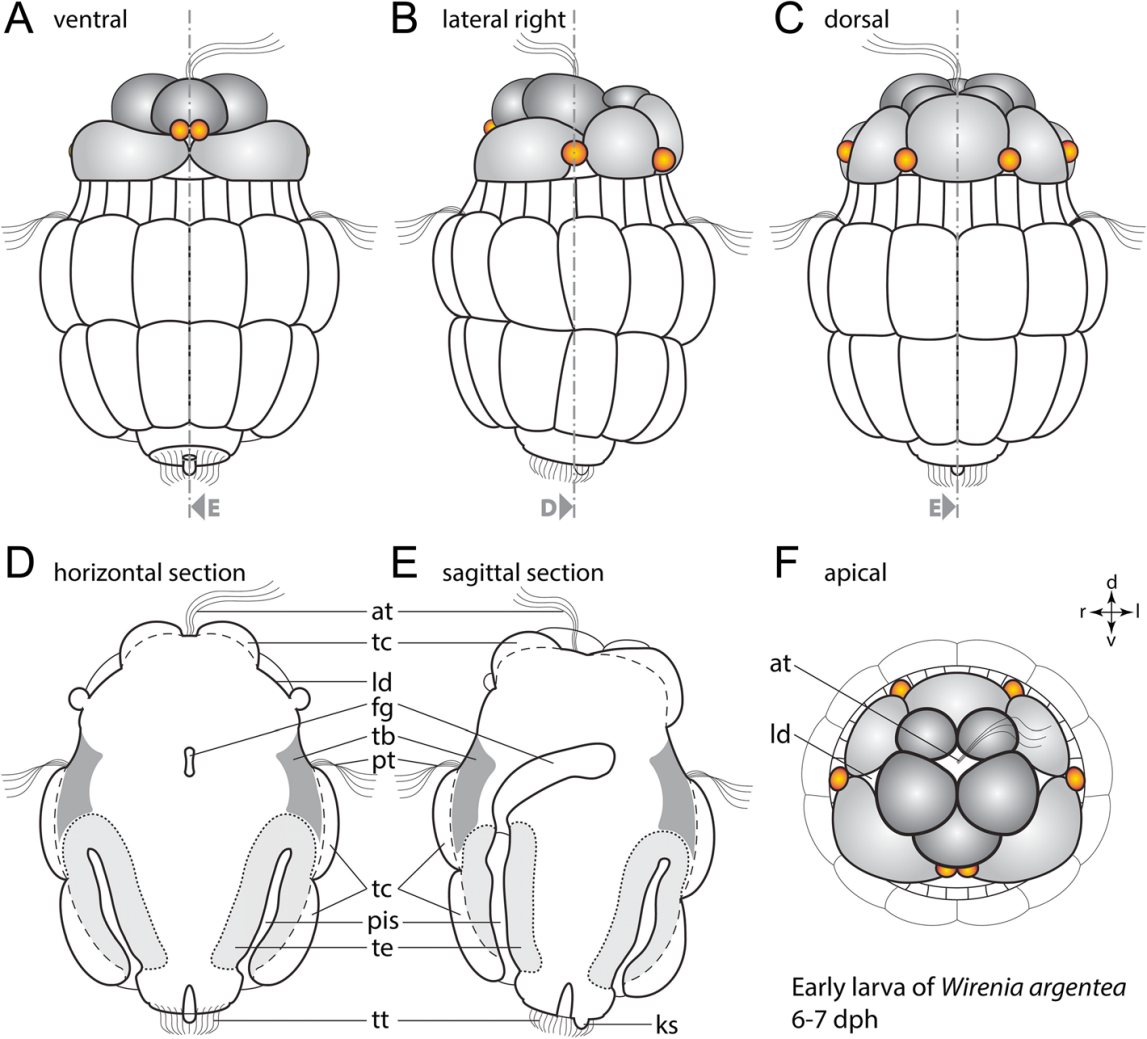
Line drawings of gross morphology of the early *W. argentea* larva. Schematic drawing of an early test cell larva (6-7 days post hatching) based on autofluorescence confocal scans. Anterior faces up in (**a**)-(**e**) and towards the viewer in (**f**). **a** Ventral view. **b** Lateral view from the right. **c** Dorsal view. **d** Horizontal section. **e** Sagittal section. **f** Anterior view of the episphere showing lateral depressions. Note the characteristic cell arrangement of two rows of test cells in the hyposphere, two rows of test cells with opposing configuration in the episphere (*grey/dark grey*), and six bilaterally arranged cells or cell protuberances (*orange*) in the episphere. Dorsal (d)–ventral (v) and left (l)–right (r) axes indicate the orientation. Abbreviations: apical tuft (at); lumen of foregut (fg); posterior knob-like structure (ks); lateral depression (ld); peri-imaginal space (pis); prototroch (pt); trochoblast (tb); test cells (tc); epidermis of the trunk (te); telotroch (tt)



*Wirenia argentea* develops via a lecithotrophic trochophore-like larva, the so-called pericalymma or test cell larva. The bell-shaped freshly hatched test cell larva features an anterior episphere with an apical tuft and a posterior hyposhere with posterior invagination, the so-called “pseudo-blastopore” [12] (Fig. 2a). The episphere is separated from the hyposphere by the ciliary prototroch formed by one row of trochoblasts (Fig. 2a). All examined larval stages of *Wirenia* reveal a characteristic cell arrangement of the covering test cells (Fig. 3; see also Additional file 1). In freshly hatched test cell larvae the posterior hyposphere is composed of two rows of test cells with two additional posteriormost and laterally positioned cells (Fig. 2a, b) [12]. The anterior episphere contains two rows of five test cells each. Both rows are arranged such that the median Z-axes of the upper cells align with the cell-cell boundary of the row below (Fig. 3a, b, c, f). Additionally, the episphere features six knob-like structures (most likely either small cells or cell protuberances), two dorsolaterally, two laterally, and two ventrally located, which are bilaterally arranged (Fig. 3f). The epispheric arrangement of bilateral knob-like structures and the two rows of test cells with opposing configuration always correlates with the ventral mouth opening of the advanced larval stages. This, in combination with the two posteriormost and laterally positioned test cells of the freshly hatched test cell larvae, allows for determining the dorso-ventral axis in larval stages that still lack significant gross morphological features such as the ventral mouth opening.

**Additional file 1:** Video of an autofluorescence scan and subsequent 3D rendering of an early test cell larva (6–7 days post hatching) of *Wirenia argentea* (Neomeniomorpha). (AVI 17546 kb)


Larval development of *Wirenia* is characterized by the outgrowth of a posterior trunk from the base of the pseudo-blastopore, resulting in a mushroom-like appearance of later stages. The first external indication of this outgrowing trunk is visible in the early test cell larva, in which the major part of the developing trunk is still masked by the outer apical cap (Fig. 3). This mode of formation results in an epidermal fold enclosing the “peri-imaginal space” (“Peri-Imaginalraum” *sensu* Salvini-Plawen [48]), which is lined by the epidermal cell layer of the outgrowing trunk on both sides (Fig. 3d, e). The early test cell larva already exhibits a ventral mouth opening and the developing foregut but lacks the two posteriormost test cells of the freshly hatched test cell larva. The typical mushroom-like appearance of later stages is recognizable in the mid-stage test cell larva, where the trunk is elongated. Late test cell larvae are already in the settlement phase and the apical cap and the covering test cells start to degenerate. The length of this phase varies individually and can last for several days [12]. At this stage, the posterior trunk is already thickened, elongated, and dorsally and laterally covered by spicules. The ventral trunk exhibits a ventrolongitudinal ciliary band, which marks the developing creeping sole.

### Gene orthologs and phylogenetic analysis

**Fig. 4:**
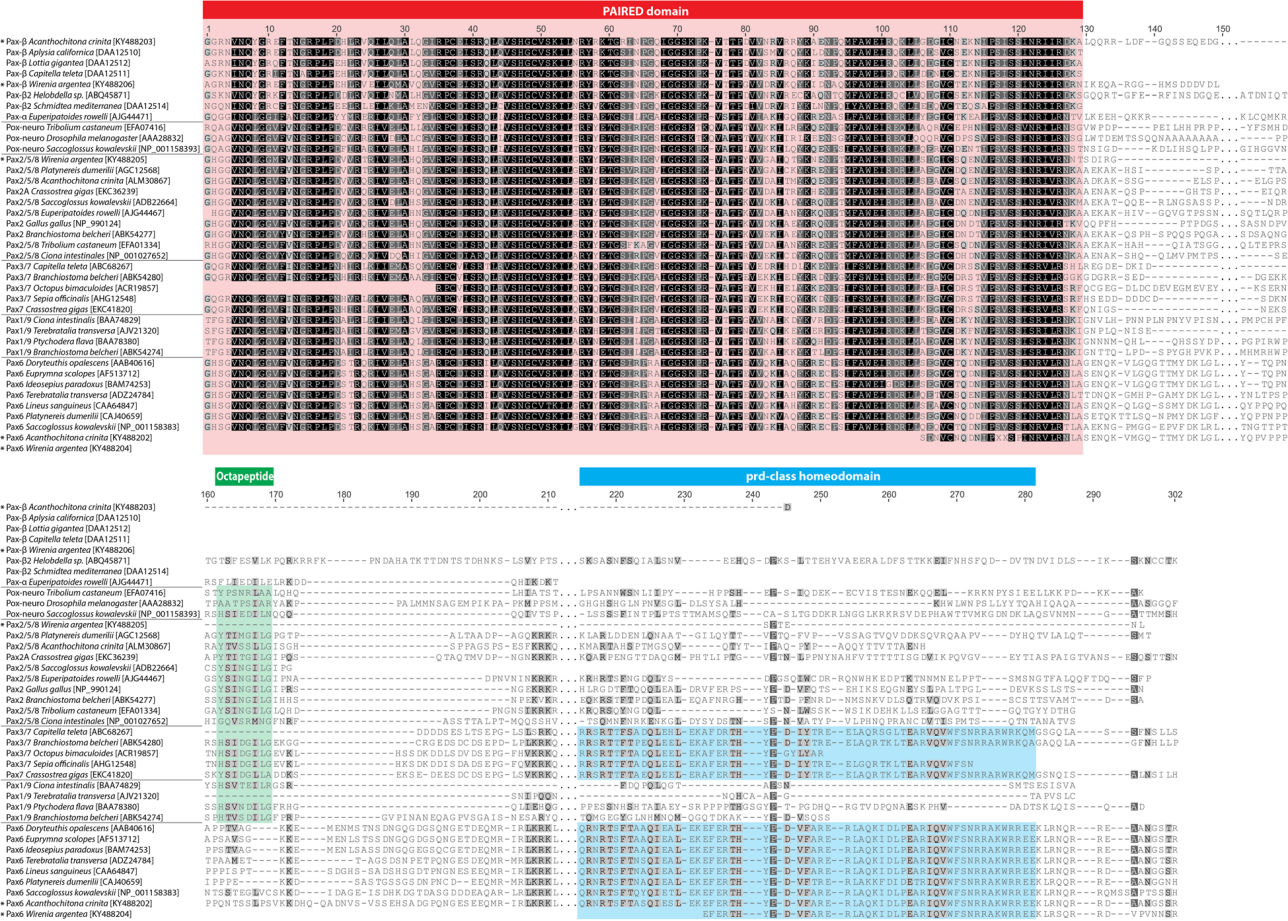
Alignment of *W. argentea* Pax genes and bilaterian orthologs. Alignment of predicted amino acid sequences of various bilaterian Pax genes including *War-Pax2/5/8, War-Pax6, War-Paxβ, Acr-Pax6* and *Acr-Paxβ* (labeled with asterisk). The conserved N-terminal PAIRED domain (*red*), and, if present, the octapeptide (*green*) and the (partial) prd-class homeodomain (*blue*) are shown, while the C-terminal transactivation domain is omitted. GenBank accession numbers of all encoding genes used are listed in Table 1

Seven bilaterian Pax groups or subfamilies have been identified by the presence or absence of highly conserved structural domains [49]. All Pax genes exhibit a conserved N-terminal Paired domain, whereas the originally existing octapeptide was lost in the paralogs *Pax4/6/10*, *Paxα/β*, and *Pax-eyg* but is still present in the paralogs *Pox-neuro*, *Pax1/9*, *Pax2/5/8*, and *Pax3/7* (Fig. 4) [50]. Another Pax gene-specific element is a so-called prd-class homeodomain, which is present in the paralogs *Pax-eyg*, *Pax3/7*, and *Pax4/6/10* as well as partially present in *Pax2/5/8* but absent in all other Pax paralogs (Fig. 4).

**Fig. 5:**
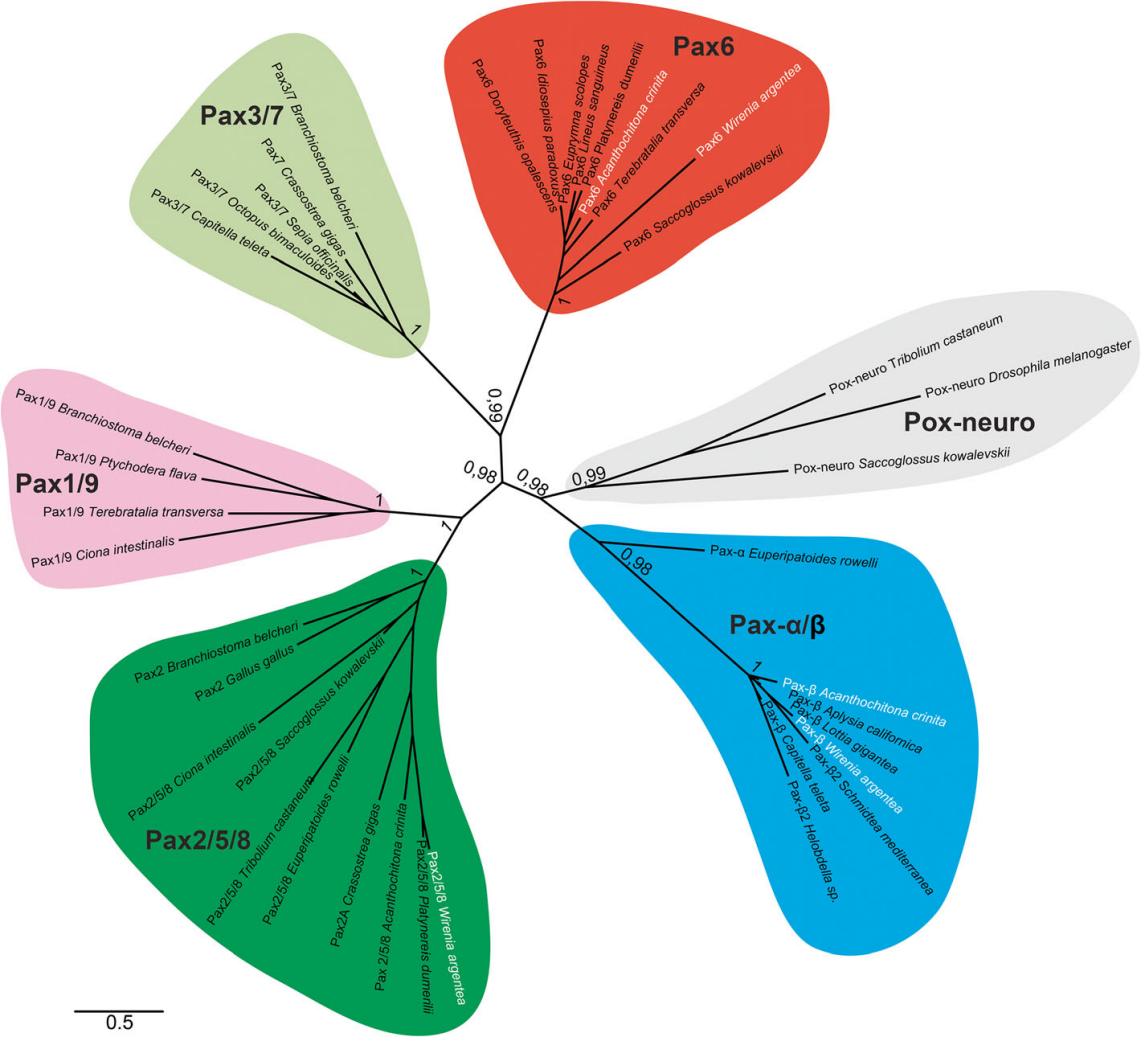
Phylogenetic reconstruction of the Pax gene family. The consensus tree was inferred through Bayesian phylogenetic analysis with MrBayes discarding 25% of samples as burn-in. The branch support values are posterior probability values of Bayesian likelihood. Pax protein families are labeled in different colors. Orthologous sequences retrieved from our transcriptomes are displayed in white letters. Note that our sequences cluster with other appropriate bilaterian orthologs

Our multiple sequence alignment includes bilaterian orthologs of all Pax subfamilies and comprised, if present, the conserved N-terminal Paired domain, the octapeptide, the prd-class homeodomain, and other conserved C-terminal domains (Fig. 4). Although our transcriptomic data revealed only partial sequences of *War-Pax6* and *Acr-Pax6*, BLAST searches against bilaterian Pax orthologs and the presence of diagnostic conserved domains (i.e., prd-class and transactivation domain) identified the two aforementioned molluscan sequences as true Pax genes (Fig. 4). The phylogenetic analysis demonstrates that all translated neomeniomorph and polyplacophoran Pax amino acid sequences (War-Pax2/5/8, War-Pax6, War-Paxβ, Acr- Pax6, Acr-Paxβ) cluster with their corresponding bilaterian orthologs (Fig. 5).

### *Pax2/5/8* expression

**Fig. 6:**
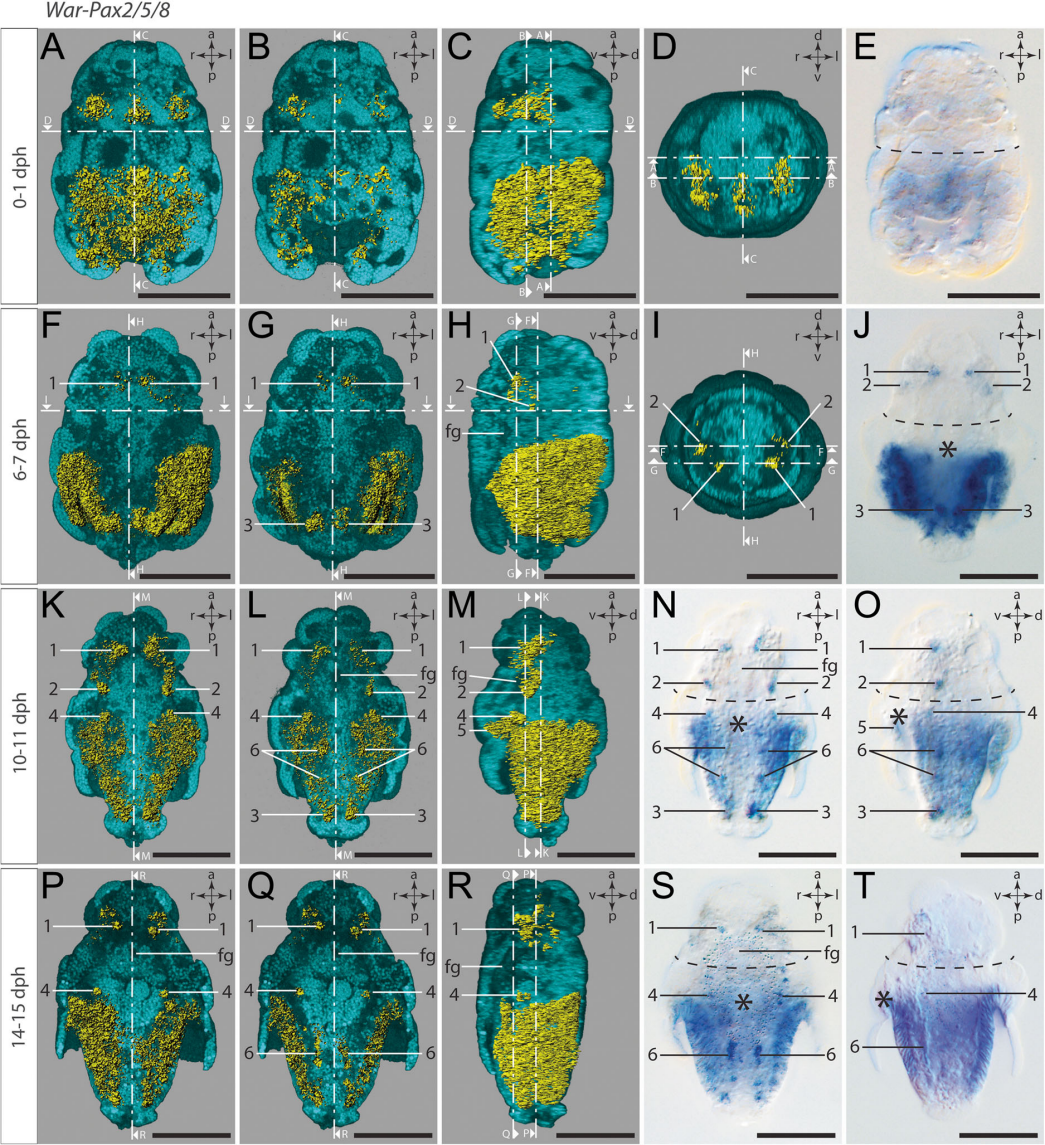
Expression of *War-Pax2/5/8.* Anterior faces up in all panels except for the anterior views in (**d**) and (**i**). Dorsal (d)–ventral (v), left (l)–right (r), and anterior (a)–posterior (p) axes indicate the orientation. First, second, and third column as well as (**d**) and (**i**) are 3D-rendered images based on autofluorescence signal (turquoise) and reflection signal of *War-Pax2/5/8* expression staining (yellow). The ventral half of the larvae in the first and second column was omitted in order to visualize the location of the ventral gene expression signal. The lateral left larval hemisphere in images of the third column was omitted in order to enable the lateral view of the gene expression signal. Clipping plane projections of the respective panels are indicated by dotted white lines. (**n**), (**s**), and fourth column are light micrographs. Location of the prototroch is indicated by dashed black lines and the location of the mouth opening is indicated by an asterisk. Numbers mark distinguishable expression domains. **a** Ventral view. **b** Ventral view with most ventrally located expression omitted. **c** Lateral left view. **d** Anterior view. **e** Ventral view. **f** Ventral view. **g** Ventral view with most ventrally located expression omitted. **h** Lateral left view. **i** Anterior view. **j** Ventral view. Note the strong expression of *War-Pax2/5/8* in the epidermal cell layer of the outgrowing trunk. **k** Ventral view. **l** Ventral view with most ventrally located expression omitted. **m** Lateral left view. **n** Ventral view. **o** Lateral left view. **p** Ventral view. **q** Ventral view with most ventrally located expression omitted. Note the expression in the epidermal cell layer of the trunk, in cells of the developing CNS (#1, #6), and in the region where the protonephridia develop (#4). **r** Lateral left view. **s** Ventral view. **t** Lateral left view. Abbreviation: foregut (fg). Scale bar: 50 μm



The free-swimming, freshly hatched test cell larvae express *War-Pax2/5/8* in three pretrochal domains, two of them bilaterally orientated and a third one located medioventrally (Fig. 6a-d; see also Additional file 2). The two bilateral domains are each expressed in a single ectodermal cell, which lies adjacent and anterior to the trochoblasts (Fig. 6b) at the base of the lateral depressions. The medioventral domain likewise lies adjacent and anterior to the trochoblasts (Fig. 6c) but at the base of a ventral depression. Additional expression of *War-Pax2/5/8* is present in ectodermal cells lining the posterior pseudo-blastopore, although the expression is weaker in the ventral ectodermal cells of the invagination (Fig. 6b, e).

**Additional file 2:** *Pax2/5/8* expression in a freshly hatched test cell larva. (AVI 25082 kb)


**Fig. 7:**
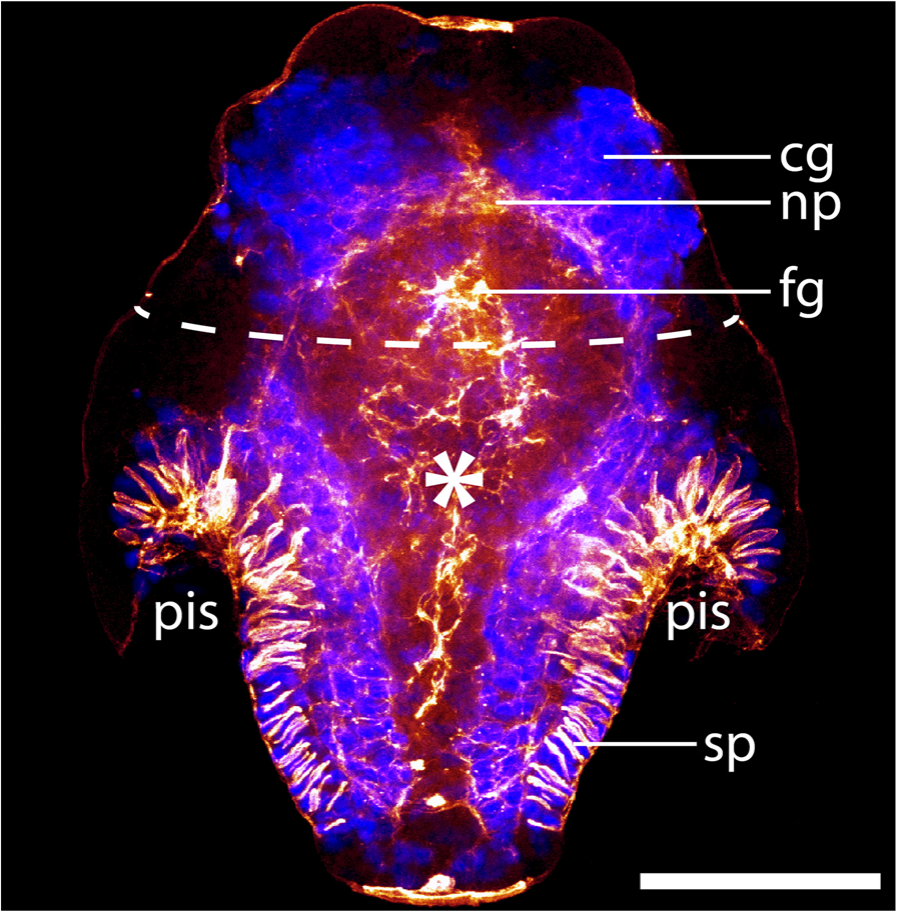
Mid-stage test cell larva. Confocal scan of a larva stained for F-actin (*red*) and DAPI (cell nuclei, *blue*). Anterior faces up. Note the staining of the F-actin-rich neuropil (np) underlying the (nuclei of the) cerebral ganglia. The dashed line indicates the prototroch. Abbreviations: nuclei of the developing cerebral ganglia (cg); developing foregut (fg); neuropil of the cerebral ganglia and cerebral commissure (np); peri-imaginal space (pis); spicule-secreting epidermal cells of the outgrowing trunk (sp); location of mouth opening (asterisk). Scale bar: 50 μm



Early test cell larvae exhibit two paired bilaterally orientated pretrochal expression domains of *War-Pax2/5/8* (Fig. 6h, i, j), which are herein referred to as “groups” (see also Additional file 3). Comparing the position of the neuropil and the corresponding nuclei of the developing cerebral ganglia (Fig. 7) with the position of both paired expression domains (Fig. 6j) shows that the anterior expression domains are located in cells or compartments of the anlagen of the cerebral ganglia. Several *War-Pax2/5/8*-expressing cells lie close to the region of the cerebral commissure (#1 in Fig. 6i, j), whereas other *War-Pax2/5/8*-expressing cells are located more laterally and adjacent to the single row of trochoblasts (#2 in Fig. 6 h, i, j). Furthermore, *War-Pax2/5/8* is expressed in the epidermal cell layer of the outgrowing trunk, which also lines the peri-imaginal space (Fig. 6f, g, h, j). This epidermal expression has a ventral gap where the future creeping sole develops. A further bilateral group of *War-Pax2/5/8*-expressing cells is located ventrally, at the posterior pole of the trunk (#3 in Fig. 6f, g, j).

**Additional file 3:** *Pax2/5/8* expression in an early test cell larva. (AVI 29128 kb)


Mid-stage test cell larvae express *War-Pax2/5/8* (Additional file 4) in cells of the anlagen of the cerebral ganglia. This cerebral expression can be subdivided in two pretrochal groups of *War-Pax2/5/8-*expressing cells. The most anterior group lies close to the cerebral commissure and adjacent and anterior to the epithelial cells of the developing foregut (#1 in Fig. 6k, l, n, o). The second group of pretrochal *War-Pax2/5/8-*expressing cells is located more posteriorly and lies adjacent to the trochoblasts (#2 in Fig. 6k-o). A third bilateral group of *War-Pax2/5/8-*expressing cells is located posttrochally and ventrally but slightly anterior to the invagination of the peri-imaginal space and the outgrowing trunk (#4 in Fig. 6k-o). Two further bilateral expression domains are located on both sides of the ventral mouth opening, which lies at the base of the invagination of the peri-imaginal space (#5 in Fig. 6o). Scattered *War-Pax2/5/8* expression is also detected in cells on both sides of and adjacent to the developing creeping sole, i.e., the region where the paired ventral nerve cords develop (#6 in Fig. 6l, n, o). As in early test cell larvae, mid-stage test cell larvae still express *War-Pax2/5/8* in the epidermal cell layer of the outgrowing trunk, which also lines the peri-imaginal space (Fig. 6k-o). A ventral gap of this epidermal expression is still present in cells of the developing creeping sole. As in early test cell larvae, a bilateral group of *War-Pax2/5/8*-expressing cells is located ventrally at the posterior pole of the trunk (#3 in Fig. 6l, n, o).



**Additional file 4:** *Pax2/5/8* expression in a mid-stage test cell larva. (AVI 25505 kb)


The pretrochal expression of *War-Pax2/5/8* in late test cell larvae (Additional file 5) is restricted to a marginal expression in the cerebral ganglia, with a more distinct expression close to the cerebral commissure (#1 in Fig. 6p, q, s). Similar to the expression pattern in mid-stage test cell larvae another bilateral expression domain of *War-Pax2/5/8* is located posttrochally and rather ventrally of the mid-horizontal section plane, and anterior to the invagination of the peri-imaginal space and the outgrowing trunk (#4 in Fig. 6p-s). As in mid-stage test cell larvae, *War-Pax2/5/8* is expressed in cells on both sides of and adjacent to the developing creeping sole, i.e., in the region where the paired ventral nerve cords are located (#6 in Fig. 6q, s). Furthermore, *War-Pax2/5/8* is still expressed in the epidermal cell layer of the outgrowing trunk (Fig. 6p-t). Thus, the ectodermal expression of *War-Pax2/5/8* in the epidermal layer of the outgrowing trunk is present throughout all developmental stages investigated (Fig. 6f, k, p). The same applies to the characteristic ventral gap of expression in the region of the developing creeping sole.



**Additional file 5:** *Pax2/5/8* expression in a late test cell larva. (AVI 29202 kb)


### *Pax6* expression

**Fig. 8:**
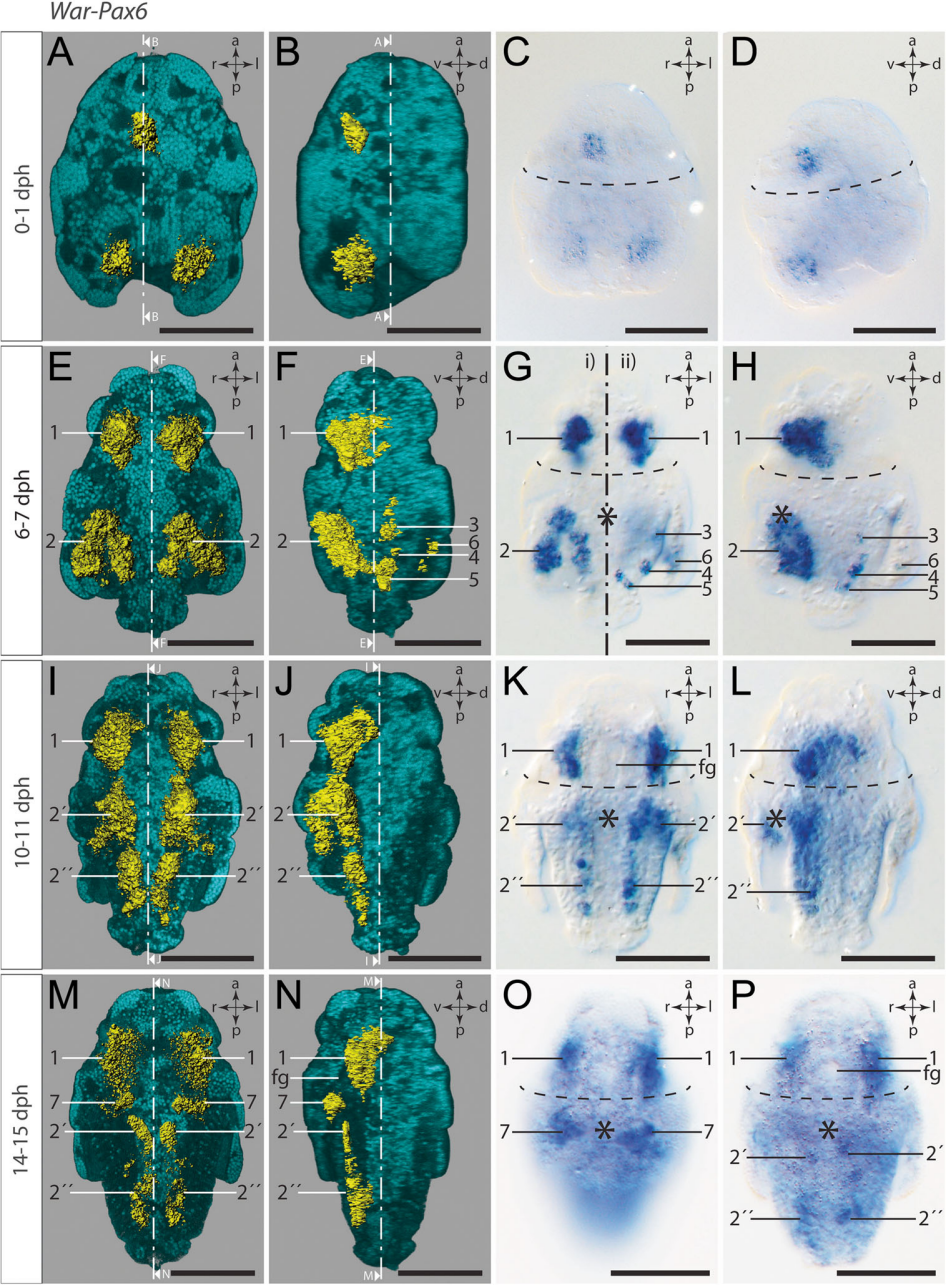
Expression of *War-Pax6.* Anterior faces up in all panels. Dorsal (d)–ventral (v) left (l)–right (r), and anterior (a)–posterior (p) axes indicate the orientation. First and second column are 3D-rendered images based on autofluorescence signal (*turquoise*) and reflection signal of *War-Pax6* expression staining (*yellow*). The ventral half of the larvae in the first column was omitted in order to visualize the location of the ventral gene expression signal. The lateral left larval hemisphere in images of the second column was omitted in order to enable a lateral view of the gene expression signal. Clipping plane projections of the respective panels are indicated by dotted white lines. Third and fourth column are light micrographs. Location of the prototroch is indicated by dashed black lines and the mouth opening by an asterisk. Numbers mark distinguishable expression domains. **a** Ventral view. **b** Lateral left view. **c** Ventral view. **d** Lateral left view. **e** Ventral view. Note the first expression signal in cells of the developing cerebral ganglia (#1). **f** Lateral left view. **g** Ventral view. i) Ventral view of ventral expression domains (#2). ii) Ventral view of dorsal expression domains (#3 - #6). **h** Lateral left view. **i** Ventral view. Note the expression in the neuroectoderm of the developing ventral nerve cords (#2´ and #2´´). **j** Lateral left view. **k** Ventral view. **l** Lateral left view. **m** Ventral view. Note *War-Pax6* expression in cells of the developing CNS, in particular in the cerebral ganglia (#1) and the ventral nerve cords (#2´ and #2´´), as well as the expression in cells flanking the mouth opening at the base of the invagination of the peri-imaginal space (#7). **n** Lateral left view. **o** Ventral view. **p** Ventral view. Note the decreasing expression in the CNS of the late larva. Abbreviation: foregut (fg). Scale bar: 50 μm



The freshly hatched test cell larvae express *War-Pax6* (Additional file 6) in a single pretrochal domain and in two posttrochal domains. The pretrochal expression of *War-Pax6* is assigned to a single medioventral cell, which lies adjacent and slightly anterior to the trochoblasts, whereas the posttrochal expression of *War-Pax6* is confined to bilaterally orientated ectodermal cells at the ventral base of the pseudo-blastopore (Fig. 8a-d).

**Additional file 6:** *Pax6* expression in a freshly hatched test cell larva. (AVI 16204 kb)


Early test cell larvae exhibit strong expression of *War-Pax6* (Additional file 7) in cells associated with the developing cerebral ganglia (#1 in Fig. 8e-h). Furthermore, strong ectodermal expression is present in two ventral stripes of epidermal cells, which line the peri-imaginal space on both sides and therefore show a hook-like appearance (#2 in Fig. 8e, f, g i, h). The outgrowing trunk exhibits three dorsal bilateral expression domains, each most likely composed of a pair of single *War-Pax6-*expressing epidermal cell (#3, #4 and #5 in Fig. 8f, g ii, h). A further paired bilateral expression domain (#6) is formed by a single cell belonging to the outer dorsolateral epidermal lining of the peri-imaginal space (Fig. 8f, g ii, h).



**Additional file 7:** *Pax6* expression in an early test cell larva. (AVI 20713 kb)


No dorsal expression of *War-Pax6* was observed in mid-stage test cell larvae. As in early test cell larvae, *War-Pax6* is strongly expressed in the anlagen of the cerebral ganglia (#1 in Fig. 8i-l; Additional file 8). Likewise, the hook-like expression domains of *War-Pax6* in the epidermal lining of the peri-imaginal space are constantly present in early and mid-stage test cell larvae (#2´ in Fig. 8i-k). Two longitudinal expression domains of *War-Pax6* (#2´´) are separated by a small gap and are situated in direct posterior extension of the hook-like domains of #2´ (Fig. 8i-l). *War-Pax6-*expressing cells of #2´´ are epidermal cells flanking the epidermal cells of the developing creeping sole (Fig. 8i, k).



**Additional file 8:** *Pax6* expression in a mid-stage test cell larva. (AVI 20261 kb)


The expression of *War-Pax6* is generally weaker in late test cell larvae, but is still present in the cerebral ganglia (#1 in Fig. 8m-p; Additional file 9). The epidermal layer with spicule-secreting cells is now restricted to the elongated trunk, which causes the longitudinal appearance of the formerly hook-like expression domain of #2´ in this late stage (Fig. 8m, n, p). *War-Pax6-*expressing cells of #2´´ are also present in late test cell larvae and, together with the cells of #2´, form two longitudinal stripes of weak expression on both sides of the developing creeping sole, i.e., in the area where the ventral nerve cords develop (Fig. 8m, n). Separated from #2´, additional *War-Pax6-*expressing cells flank the ventral mouth opening at the base of the invagination of the peri-imaginal space (#7 in Fig. 8m, n, o).



**Additional file 9:** *Pax6* expression in a late test cell larva. (AVI 19856 kb)


### *Paxβ* expression

**Fig. 9:**
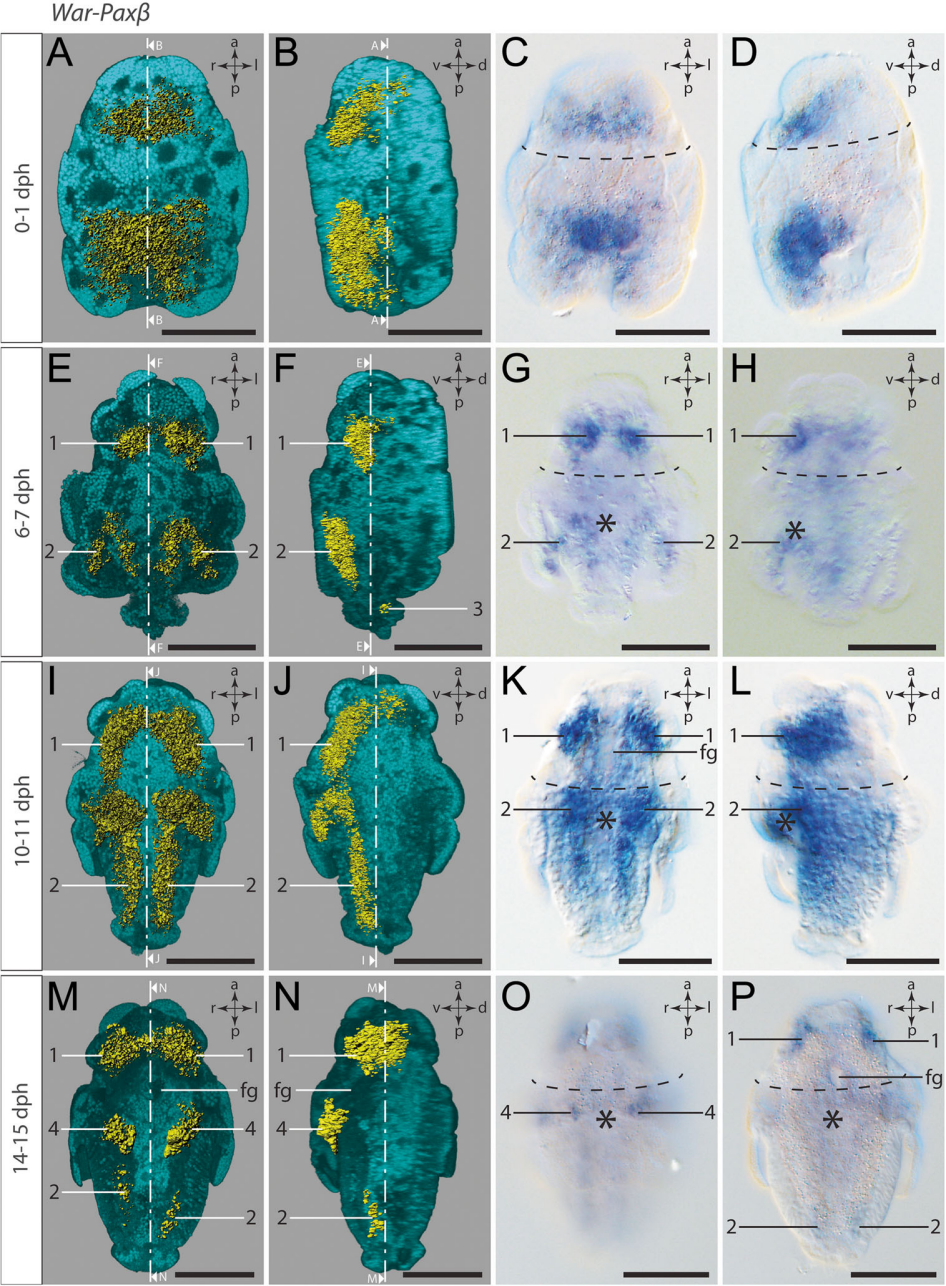
Expression of *War-Paxβ.* Anterior faces up in all panels. Dorsal (d)–ventral (v) left (l)–right (r), and anterior (a)–posterior (p) axes indicate the orientation. First and second column are 3D rendered images based on autofluorescence (turquoise) and reflection signal of *War-Paxβ* expression staining (*yellow*). The ventral half of the larvae in the first column was omitted in order to visualize the location of the ventral gene expression signal. The lateral left larval hemisphere in images of the second column was omitted in order to enable a lateral view of the gene expression signal. Clipping plane projections of the respective panels are indicated by dotted white lines. Third and fourth column are light micrographs. Location of the prototroch is indicated by dashed black lines and the location of the mouth opening is indicated by an asterisk. Numbers mark distinguishable expression domains. **a** Ventral view. **b** Lateral left view. **c** Ventral view. **d** Lateral left view. **e** Ventral view. Note the first expression signal in cells of the developing cerebral ganglia (#1). **f** Lateral left view. **g** Ventral view. **h** Lateral left view. **I** Ventral view. Note the expression in the neuroectoderm of the developing ventral nerve cords (#2) and cerebral ganglia (#1). **j** Lateral left view. **k** Ventral view. **l** Lateral left view. **m** Ventral view. Note *War-Paxβ* expression in cells of the cerebral ganglia (#1) and the faint expression in the region of the developing ventral nerve cords (#2) as well as the expression in cells flanking the mouth opening at the base of the invagination of the peri-imaginal space (#4). **n** Lateral left view. **o** Ventral view of the most ventral part of a late larva. **p** Ventral view. Note the weak *War-Paxβ* expression in the CNS (#1, #2). Abbreviation: foregut (fg). Scale bar: 50 μm



The expression pattern of *War-Paxβ* is largely consistent with the corresponding expression of *War-Pax6*. Freshly hatched test cell larvae express *War-Paxβ* in a single pretrochal domain in the same medioventral region adjacent to the ventral trochoblasts that also shows *War-Pax6* expression; however, the *War-Paxβ* expression domain is wider and encompasses more than one single cell (Fig. 9a-d, Additional file 10). Besides the pretrochal expression, freshly hatched test cell larvae also exhibit posttrochal expression in epidermal cells located ventrally of the pseudo-blastopore (Fig. 9a-d).

**Additional file 10:** *Paxβ* expression in a freshly hatched test cell larva. (AVI 30190 kb)


As is the case for *War-Pax6,* early test cell larvae express *War-Paxβ* (Additional file 11) in cells of the developing cerebral ganglia (#1) as well as in two ventral stripes of epidermal cells, which line the peri-imaginal space on both sides and therefore show a hook-like appearance (#2 in Fig. 9e-h). Furthermore, *War-Paxβ* is expressed in two rather dorsally located spots at the posterior pole of the outgrowing trunk (#3 in Fig. 9f). Each spot is most likely formed by a single *War-Paxβ-*expressing cell.



**Additional file 11:** *Paxβ* expression in an early test cell larva. (AVI 18566 kb)


In mid-stage test cell larvae *War-Paxβ-*expressing cell groups are located in similar expression domains as described for the early test cell larvae and thus closely resemble the *War-Pax6* expression pattern of mid-stage test cell larvae (Additional file 12). Strong *War-Paxβ* expression is present in the anlagen of the cerebral ganglia (#1) and in two ventral stripes of ectodermal cells adjacent to the cells of the developing creeping sole (#2 in Fig. 9i-l). As is the case for the *War-Pax6* expression, the stripes of *War-Paxβ-*expressing ectodermal cells of group 2 also line the fold of the peri-imaginal space, which causes a hook-like appearance at the anterior pole of the expression domain.



**Additional file 12:** *Paxβ* expression in a mid-stage test cell larva. (AVI 16661 kb)


Expression of *War-Paxβ* in late test cell larvae (Additional file 1) is comparatively low. The most intense expression is still present in the developing cerebral ganglia (#1 in Fig. 9m, n, p), which is congruent with the expression of *War-Pax6* in late test cell larvae. A further analogy to the expression pattern of *War-Pax6* is the location of two spots of *War-Paxβ-*expressing cells flanking the mouth opening at the base of the invagination of the peri-imaginal space (#4 in Fig. 9m, n, o). The ventral stripe-like *War-Paxβ* expression on both sides adjacent to the creeping sole (#2; similar to the younger stages) is hardly detectable in late test cell larvae and, if visible, is mostly limited to a faint expression in the ventral part of the trunk (#2 in Fig. 9m, n, p).



**Additional file 13:** *Paxβ* expression in a late test cell larva. (AVI 11934 kb)


## Discussion

### Putative ancestral and co-opted roles of *Pax2/5/8* in neomeniomorph mollusks

*Pax2/5/8* is an ortholog of the *Drosophila Sv/Spa* and the vertebrate *Pax2*, *Pax5,* and *Pax8* genes. These play a crucial role in the development and regionalization of the highly centralized and complex brains of vertebrates and insects as well as cephalopods, where they were most likely independently recruited into similar functions [24, 27]. Based on its expression in sensory structures of chordates (e.g., [23, 51]), insects [52], and mollusks [25], *Pax2/5/8* was proposed to have a conserved role in the formation of structures responsible for balance and geotaxis in eumetazoans [25].

For Mollusca, our data on an aplacophoran supplement existing *Pax2/5/8* expression data on polyplacophorans, gastropods, bivalves, and cephalopods [25, 27, 53]. Although *Pax2/5/8* was found to be expressed in the adult brain of the gastropod *Haliotis* [53], there is no evidence that this gene is involved in its development [25]. However, developmental expression of *Pax2/5/8* in *Haliotis* was found in the statocysts, eyes, foot, and in putative chemosensory organs of the pallial chamber [25]. The expression of *Pax2/5/8* in putative (precursors of) sensory cells was also confirmed for a polyplacophoran, where it is probably expressed in the developing esthetes (shell eyes) and the larval ampullary system (cf. [54]). Though, it was proposed that *Pax2/5/8* expression probably predates sensory cell development in multimodal sensory systems during molluscan ontogeny [27]. Although neomeniomorphs do possess distinct sensory organs, such as the atrial sense organ or the dorsoterminal sense organ, we did not find any evidence for *Pax2/5/8* expression in cells that could be unambiguously assigned to a distinct sensory structure. However, *Wirenia* exhibits *Pax2/5/8* expression in cells dedicated to the developing cerebral ganglia and the ventral nerve cords, which is in stark contrast to the other investigated molluscan representatives with a considerable simple (i.e., little concentrated) CNS, such as bivalves, gastropods, and polyplacophorans, but is in accordance with *Pax2/5/8* expression in the highly centralized brain of cephalopods. Since data for only one species per class-level taxon are currently available, more comparative analyses are required to further interpret this finding. Nevertheless, and in contrast to *War-Pax6* and *War-Paxβ, War-Pax2/5/8* is not equally expressed in the region of the developing cerebral ganglia, but instead shows increased expression in cells adjacent to the apical organ and the cerebral commissure (#1 in Fig. 6 f-t), as well as in a slightly more posterior location where the future pedal ganglia develop (#2 in Fig. 6 f-o). Although neomeniomorphs do not exhibit a highly centralized CNS, this specific *Pax2/5/8* expression could hint towards a similar function in CNS regionalization comparable to its proposed function in cephalopods (cf. [27]).

Aside from its putative function in patterning neural or sensory structures, *Pax2/5/8* is also expressed during development of excretory organs, such as the nephridia of onychophorans and annelids [26, 35] and the kidneys of vertebrates [55, 56]. Interestingly, *Wirenia* is the only investigated mollusk, which shows *Pax2/5/8* expression in the region where the paired protonephridia of the larva develop (#4 in Fig. 6 k-t, see also [12]). Whether this hints towards a conserved function of *Pax2/5/8* in bilaterian nephrogenesis or whether this represents a coopted function of the aplacophoran lineage remains unclear until more data on various clades become available.

Remarkably, *Pax2/5/8* is expressed in epidermal spicule-secreting or associated cells of neomeniomorph as well as polyplacophoran mollusks [27]. Although it was not explicitly mentioned for Polyplacophora, the respective light micrographs show a distinct expression signal in the spicule-secreting perinotum (or girdle) of *Acanthochitona crinita* (cf. Fig. 6 g in [27]). Not much is known about spicule secretion and its molecular background in aculiferan mollusks, but a recent study on the evolution of molluscan secretomes and biomineralization processes proposed that independent co-option of gene families are important driving forces acting on molluscan biomineralization [57]. Since the secretion of calcareous spicules most likely constitutes an aculiferan autapomorphy and *Pax2/5/8* appears not to be involved in molluscan shell development [25, 27], it seems likely that *Pax2/5/8* expression in the spicule-secreting tissues of Polyplacophora and Neomeniomorpha evolved along the line leading towards the Aculifera after their split from their conchiferan allies.

### Conserved *Pax6* expression in aculiferan molluscs

*Pax6* is generally expressed in developing nervous systems and in various photosensory organs, regardless of their overall morphology in numerous metazoans, which underlines its important role as regulatory gene in eye developmental networks (see, e.g., [58–66]). Prior to our study, molluscan *Pax6* expression had only been studied in developmental stages of cephalopods [30, 31, 67, 68] and a polyplacophoran [32], as well as in an adult gastropod [53]. The latter study showed that neural expression of *Pax6* in adults of the gastropod *Haliotis asinina* is in the cerebral and pleuropedal ganglia as well as in sensory structures such as the eyes, tentacles, and gills [53]. Cephalopod developmental *Pax6* expression in the squids *Loligo opalescens* and *Euprymna scolopes* is confined to their brain lobes, eyes, and olfactory organ [30, 67]. The polyplacophoran *Leptochiton asellus* likewise shows *Pax6* expression in the developing nervous system as well as in the ventrally and posttrochally positioned larval eyes [32]. Since the latter study largely focused on eye development, we analyzed *Pax6* expression during neurogenesis of another polyplacophoran representative, *Acanthochitona crinita* (unpublished data). Our data confirm *Pax6* expression in the region of the developing ventral nerve cords but not in the lateral nerve cords of the tetraneural nervous system of this polyplacophoran species. Interestingly, this finding is congruent with *Pax6* expression in later developmental stages of the neomeniomorph *Wirenia argentea,* which argues for a conserved expression of this gene during neurogenesis of the ventral nerve cords of protostomes (cf., e.g., data on various crustaceans, insects, onychophorans, planarians, annelids, and polyplacophorans) [32, 62, 63, 66, 69–71]. Moreover, a conserved bilaterian mode of expression of *Pax6* is also present in the larval episphere of *W. argentea*, where it correlates with the development of the cerebral ganglia.

Freshly hatched test cell larvae of *Wirenia* express *Pax6* in two patches at the ventral base of the pseudo-blastopore, i.e., in the region where a pair of neurite bundles emerges from the posterior neurogenic domain (cf. [13]). Cells of the apical organ, the only larval sensory organ known from neomeniomorphs, do not exhibit *Pax6* expression. The most complex *Pax6* expression pattern occurs in early test cell larvae of *Wirenia* (#3, #4, #5, and #6 in Fig. 8f, g ii, h). Since this expression pattern is neither complemented by coexpression of *Pax2/5/8* or *Paxβ* nor supported by neurotransmitter distribution of 5-HT (serotonin) and FMRF-amide, the neurogenic nature of these *Pax6* expressing cells is questionable. Remarkably, late test cell larvae of *Wirenia* exhibit two patches of *Pax6*-expressing cells at the anterior margin of the larval hyposphere and lateral to the ventral mouth opening, which is congruent with two patches of *Paxβ*-expressing cells (compare #7 in Fig. 8m, n, o and #4 in Fig. 9m, n, o). By comparing the polyplacophoran trochophore larva with the neomeniomorphan test cell larva we found that this specific location of the *Pax6-* and *Paxβ*-expressing cells correlates with the location of the polyplacophoran larval eyes and its associated *Pax6* expression. Although, as far as currently known, neomeniomorph larvae and adults lack distinct photosensory organs (eyes), this correlation and the proposed close relationship of Polyplacophora and Neomeniomorpha calls for further morphological investigations combined with expression studies of suitable candidate genes that should focus on putative (rudimentary?) photoreceptors in this region.

### The putative role of Paxα/β during bilaterian development

The Paxα/β group is the most recently discovered Pax subfamily and has tentatively been named “Pax?” [34]. Initially, sequences of the “Pax?” subfamily have been shown to be present in Ctenophora, Deuterostomia (Echinodermata, Hemichordata), Nematoda, Annelida, and Platyhelminthes [34]. Another study revealed a lophotrochozoan-specific Paxβ clade [33], which could also be assigned to the “Pax?” assemblage. Recently, a Paxα clade was described as supposed sister group to the lophotrochozoan-specific Paxβ clade [35]. The Paxα clade comprises sequences of Panarthropoda as well as Deuterostomia, whereas the previously included ctenophore PaxA/B sequences received less support. Therefore, “Pax?” is considered a distinct bilaterian Pax subfamily termed “Paxα/β“ [35]. The ancestral role of the Paxα/β subfamily in bilaterians remains unknown. However, the *Paxβ* expression data of the single investigated lophotrochozoan so far, the leech *Helobdella*, reveal expression of two *Paxβ* homologs, *Hsp-Paxβ1* and *Hsp-Paxβ2*, in the developing CNS and, in case of *Paxβ1,* additionally at the site of eye formation [33]. A similar situation was found in the onychophoran *Euperipatoides rowelli,* where *Ero-Paxα* is expressed in the CNS, more precisely in the lateral brain tissue associated with each eye of the adult animal [35]. These findings are now complemented by our data on the expression of *War*-*Paxβ* in the region of the developing cerebral ganglia and the ventral nerve cords of the neomeniomorph mollusk *W. argentea*. Although the role of *Paxα/β* is obviously not exclusively assigned to developmental processes, the common expression in the CNS of all species hitherto investigated argues for an ancestral role in CNS specification at least in protostomes. Since data of *Paxα* expression in Deuterostomia are still lacking, the ancestral role of the Paxα/β subfamily for the entire Bilateria remains obscure. However, both investigated lophotrochozoans, *W. argentea* and *Helobdella austinensis*, show distinct *Paxβ* expression in the CNS of their anterior (“head”) region as well as in their ventral nerve cords (present study, [33]). The results for *W. argentea* are in accordance with the findings in another aculiferan mollusk, the polyplacophoran *Acanthochitona crinita*, where it is expressed in the lateral region of the cerebral commissure and in the ventral nerve cords (unpublished data). These congruent expression patterns in *H. austinensis*, *A. crinita*, and *W. argentea* argue for a conserved expression of *Paxβ* during neurogenesis of the ventral nerve cords of annelids and (aculiferan) mollusks.

### Expression of *Pax2/5/8, Pax6,* and *Paxβ* and homology of lophotrochozoan ventral nerve cords

**Fig. 10:**
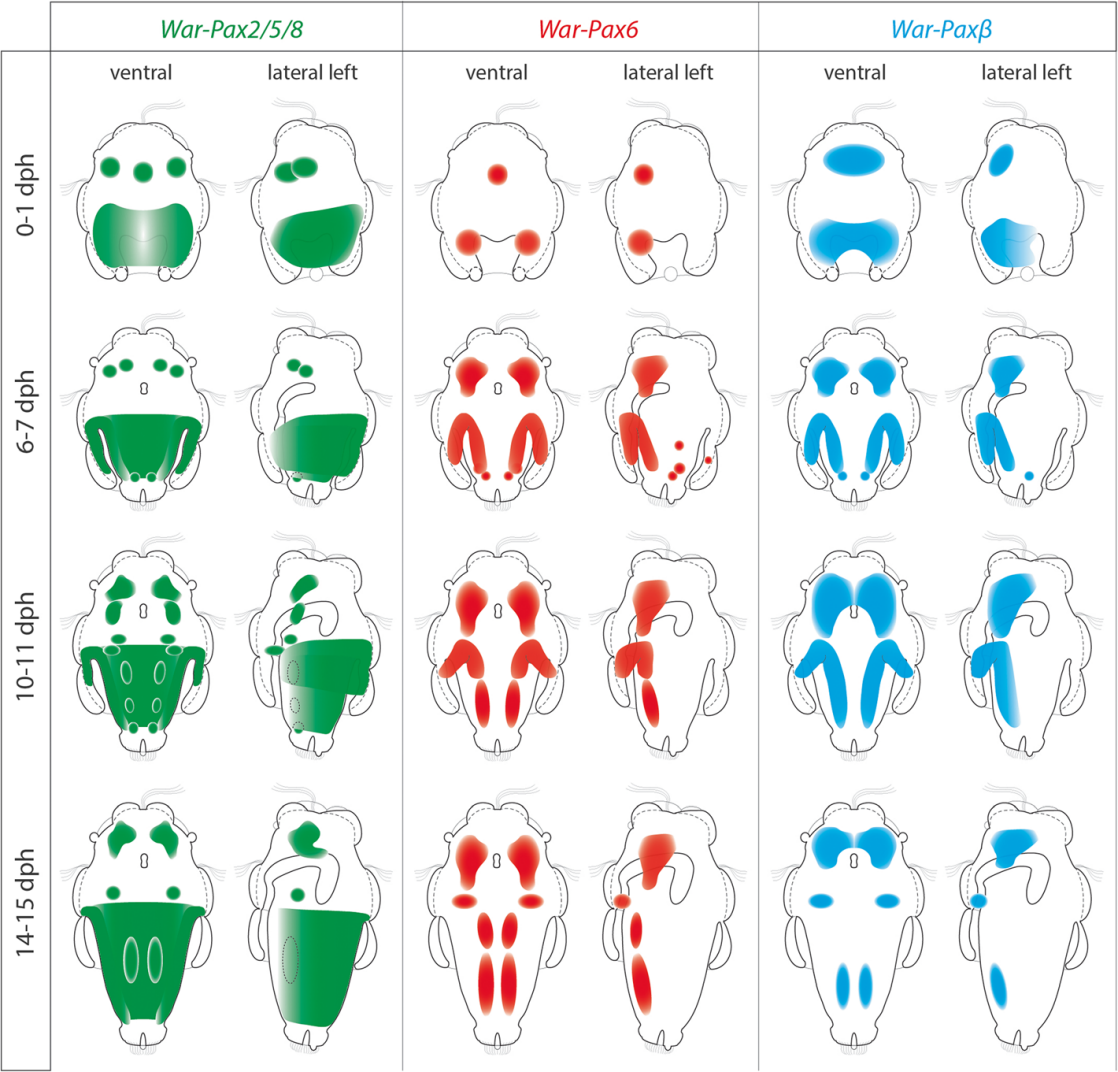
Schematic summary of Pax gene expression in *Wirenia argentea*

Among other components, the molluscan nervous system is generally composed of an esophageal nerve ring (including a paired cerebral ganglion) and a paired pedal ganglion. From the former, two lateral (visceral) nerve cords emanate, while the latter gives rise to a pair of ventral (pedal) nerve cords. Together, these four longitudinal nerve cords form the tetraneural nervous system of Mollusca [7, 8]. The expression of *Paxβ*, *Pax6,* and *Pax2/5/8* in anterior and posterior domains of early developmental stages of *Wirenia argentea* (Fig. 10) reflects the morphogenetic data that show that neurogenesis starts with the outgrowth of a pair of neurite bundles from both the neuropil of the apical organ and a posterior neurogenic domain [13]. Interestingly, none of the three genes investigated herein appears to be expressed in the developing lateral nerve cords of *W. argentea,* but are so in the developing ventral nerve cords (Fig. 10). This is congruent with *Paxβ* and *Pax6* expression in the polyplacophorans *Acanthochitona crinita* (unpublished data) and – at least for *Pax6* expression – *Leptochiton asellus* [32]. Interestingly, annelids likewise show expression of *Pax2/5/8*, *Pax6*, and *Paxβ* in the ventral nerve cords [26, 33, 62, 63]. This shared expression domain in the paired ventral nerve cord of annelids and neomeniomorphs strongly suggests a common evolutionary origin and thus argues for homology of ventral nerve cords in the lophotrochozoan lineage, thus supporting the notion that one pair of ventral nerve cords was present in the last common lophotrochozoan ancestor (see also [7]).

## Conclusion

Our data provide first insights into the molecular background of aplacophoran neurogenesis on gene expression level and thus add significant insight into the putative roles of the herein investigated Pax genes in molluscan development. The *Pax6* expression pattern in the aculiferan clades Neomeniomorpha and Polyplacophora largely resembles the common bilaterian expression during CNS development. The expression of *Paxβ* in the CNS of the neomeniomorph *Wirenia argentea* and the leech *Helobdella austinensis* argues for an ancestral role in lophotrochozoan neurogenesis, in particular during formation of the cerebral ganglia and the ventral nerve cords. Furthermore, we found indication for a conserved role of *Pax2/5/8* in CNS development in Bilateria, while its expression in the spicule-secreting or associated cells in both neomeniomorphs and polyplacophorans suggests a novel function of this gene in aculiferan skeletogenesis. None of the investigated Pax genes are involved in the development of the lateral (visceral) nerve cords in either Neomeniomorpha or Polyplacophora, suggesting a different molecular background of this tetraneural subset on the one hand and homology of the ventral nerve cords within Spiralia (or Lophotrochozoa) on the other.
